# Therapy-Driven Molecular Evolution of Bladder Cancer: Roles of Cellular Plasticity and Tumor Microenvironment

**DOI:** 10.3390/ijms27125152

**Published:** 2026-06-06

**Authors:** Seung-Woo Baek, Seo-Young Yoon, Seon-Kyu Kim, Sun-Hee Leem

**Affiliations:** 1 Genomic Medicine Research Center, Korea Research Institute of Bioscience and Biotechnology, Daejeon 34141, Republic of Korea; baek@kribb.re.kr; 2 Department of Biomedical Sciences, Dong-A University, Busan 49315, Republic of Korea; ysy05166@hanmail.net; 3 Department of Health Sciences, The Graduated of Dong-A University, Busan 49315, Republic of Korea; 4 Department of Bioscience, University of Science and Technology, Daejeon 34141, Republic of Korea

**Keywords:** drug resistance, non-genetic plasticity, metabolic rewiring, tumor microenvironment, chemoresistance, molecular evolution

## Abstract

Drug resistance remains a significant barrier to achieving durable treatment responses. Traditionally, resistance has been attributed to genetic alterations and clonal selection. However, accumulating evidence suggests that early adaptation to therapy is often mediated by non-genetic state transitions. In this review, we propose a conceptual framework in which resistance emerges through therapy-driven molecular evolution in bladder cancer, characterized by three interconnected axes: non-genetic plasticity, metabolic reorganization, and tumor microenvironment remodeling. Using the Gemcitabine-Resistant Cell (GRC) model as a temporal reference system, we describe a stepwise transition from drug-sensitive states dominated by proliferation to survival-optimized resistant states through a growth–survival trade-off. Early adaptive phases are marked by the attenuation of cell-cycle and glycolytic programs, increased epigenetic flexibility, and metabolic rewiring involving mitochondrial and lipid-associated pathways. Later phases involve the reinforcement of resistance through extracellular matrix remodeling, developmental and stress-response signaling, and immunometabolic interactions within the tumor microenvironment, including adenosine- and lipid-associated mediators. Projecting the GRC score onto a clinical bladder cancer cohort further suggests that these evolutionary patterns may also be reflected in patient tumors. Overall, this framework supports a temporally structured view of chemoresistance and highlights opportunities to therapeutically target transitional adaptive states before resistance becomes stabilized.

## 1. Introduction

Cancer cells are continuously exposed to fluctuating environmental pressures, such as nutrient limitation, hypoxia, immune surveillance, and therapeutic intervention. Among these factors, anticancer therapy stands out as one of the most intense and sustained forms of external stress [1,2]. Rather than acting solely as a cytotoxic agent, therapy serves as a powerful evolutionary force that reshapes the tumor ecosystem and selectively favors cells that are capable of adaptive survival [3]. In this context, drug resistance should not be viewed merely as the consequence of discrete genetic mutations but as the outcome of stress-induced molecular evolution.

Traditionally, resistance has been explained by the selection of pre-existing mutant clones or the emergence of de novo genomic alterations. While genetic selection certainly plays a role in late-stage refractory disease, accumulating evidence suggests that the earliest adaptive responses to therapeutic stress are primarily non-genetic [2,4]. Upon exposure to cytotoxic agents like gemcitabine, tumor cells quickly reprogram their transcriptional networks, metabolic processes, and chromatin states, all without immediate changes to their DNA sequence [5,6]. These reversible alterations provide temporary survival advantages, allowing a subset of cells to endure otherwise lethal conditions. This stress-induced acquisition of non-genetic traits exemplifies a form of adaptive plasticity that precedes and facilitates genetic fixation. By entering alternative functional states, such as reduced proliferation, metabolic downshift, or enhanced antioxidant defense, cells temporarily sacrifice growth potential to maintain viability [6,7]. Such adaptations expand the evolutionary search space, enabling tumor populations to explore various survival configurations under sustained selective pressure. Over time, persistent stress stabilizes these adaptive states through epigenetic reinforcement, signaling network consolidation, and eventual genomic remodeling, transforming reversible plasticity into a structurally embedded form of resistance [2,6,7].

Importantly, therapy-induced molecular evolution does not occur in isolation within tumor cells. Simultaneously, external stress remodels the tumor microenvironment (TME), altering cytokine secretion, extracellular matrix composition, metabolic byproducts, and immune cell recruitment [3,8,9,10,11,12,13,14]. These environmental changes create new selective gradients that further refine clonal fitness, leading to the emergence of resistance through the co-evolution of tumor-intrinsic adaptive programs and stress-modified ecological niches.

The Gemcitabine-Resistant Cell (GRC) model offers a valuable framework for exploring this evolutionary trajectory [15,16].

In this review, we utilize previously published experimental models not as new data but as temporal reference systems to organize the broader concept of therapy-driven molecular evolution. By tracking sequential passages (P0 → P3 → P7 → P15) under sustained drug exposure, we can observe the gradual transition from early survival adaptation to stable resistance. This temporal framework reveals a growth–survival trade-off: tumor cells initially downregulate proliferative programs to activate defense-oriented metabolic and signaling pathways, followed by a gradual re-engagement of proliferative capacity once adaptive stability is achieved.

We conceptualize drug resistance as a staged process of external stress-driven molecular evolution in bladder cancer. We begin by outlining the dynamic trajectory observed in the GRC model and introducing the growth–survival trade-off as a key organizing principle. We then analyze the early acquisition of non-genetic plasticity, including metabolic reorganization and epigenetic remodeling, before examining how later-stage resistance is reinforced through signaling interactions and secreted factors within the tumor microenvironment. Finally, we discuss therapeutic strategies aimed at interrupting or redirecting this evolutionary process before adaptive states become irreversibly fixed.

By integrating external stress, the acquisition of non-genetic traits, and time-resolved molecular rewiring into a unified framework, this perspective shifts the focus from static resistance markers to the evolutionary dynamics that produce them.

## 2. Therapies as an External Selective Pressure and Trigger of Molecular Evolution

### 2.1. Therapies as an External Selective Pressure and Evolutionary Trigger

Modern oncology is anchored by three pillars: cytotoxic chemotherapy, molecularly targeted therapy, and immune checkpoint inhibitors (ICIs) [17,18]. While these modalities diverge in their molecular targets and clinical applications, they converge on a singular biological consequence: the imposition of strong external selection pressures that radically alter the fitness landscape of the tumor ecosystem. Cytotoxic chemotherapy, including platinum agents (e.g., cisplatin), antimetabolites (e.g., gemcitabine, 5-FU), and microtubule-targeting agents (e.g., taxanes), primarily disrupts DNA integrity and mitotic machinery. Although their mechanisms vary, their ecological outcome is the same: the selective elimination of rapidly proliferating cells. This bottleneck favors subpopulations that can enter quiescence, enhance DNA repair, or tolerate replicative stress. Consequently, resistance often emerges not through novel mutations but through adaptive transitions toward slow-cycling or metabolically reprogrammed states [19,20]. Molecularly targeted therapies impose selective constraints by specifically inhibiting oncogenic signaling pathways. For example, EGFR inhibitors (e.g., osimertinib), MAPK pathway blockers (e.g., BRAF/MEK inhibitors), and anti-angiogenic agents (e.g., bevacizumab) reconfigure the tumor’s signaling architecture. However, resistance often develops due to secondary mutations, activation of bypass pathways, or lineage plasticity. This selection pressure favors signal redundancy and phenotypic switching, enabling cells that can rewire their intracellular networks to survive despite ongoing pathway suppression [18,21,22,23,24]. Immune checkpoint inhibitors (targeting PD-1/PD-L1 or CTLA-4) impose a distinct evolutionary constraint: immune visibility [25,26]. Unlike direct inhibitors, ICIs restore the host’s anti-tumor activity. As a result, resistance develops through the loss of antigen presentation machinery, defects in interferon signaling, or the recruitment of immunosuppressive populations within the tumor microenvironment (TME) [27,28]. This results in the emergence of immune-evasive phenotypes instead of resistance driven solely by proliferation. In summary, cancer therapy should be seen as a dynamic environmental pressure rather than a fixed intervention. Each treatment modality acts as an ecological bottleneck that reshapes tumor heterogeneity: cytotoxic agents promote stress tolerance, targeted therapies enhance network plasticity, and immunotherapy encourages immune evasion. Collectively, these treatments influence the evolutionary trajectory of the tumor ecosystem. Cancer is increasingly viewed as an evolutionary system governed by the principles of variation, selection, and inheritance. Within this framework, anticancer therapy acts like an environmental bottleneck, significantly altering fitness landscapes and reshaping clonal architecture. During treatment, sensitive clones are eliminated or experience growth arrest, while subpopulations with genetic or non-genetic survival advantages endure. This selective process mirrors Darwinian selection, where treatment imposes directional pressure favoring phenotypes that can survive the drug. Importantly, growing evidence indicates that therapy not only selects for pre-existing resistant mutations but can also actively induce adaptive state transitions.

Russo et al. (2024) demonstrated the emergence of drug-tolerant persister (DTP) cells, which are characterized by reversible chromatin-mediated transcriptional reprogramming [29,30,31,32,33,34]. These findings indicate that non-genetic heterogeneity allows for transient survival states that precede stable resistance. Recent single-cell analyses further support this dynamic perspective [29,35,36,37,38]. Shaffer et al. (2017) showed that rare transcriptional fluctuations can prime cells for survival under targeted therapy, and subsequent drug exposure stabilizes these states [1,39,40,41,42]. Thus, therapy serves not only as a filter but also as a catalyst that amplifies phenotypic variability. In this context, anticancer therapy should be viewed as a powerful evolutionary trigger that restructures tumor ecosystems over time. Consequently, resistance emerges as a trajectory rather than a discrete event.

### 2.2. Mechanistic Axes of Therapy-Driven Molecular Evolution

Therapy-induced molecular evolution occurs through multiple interconnected mechanisms. While genetic mutations contribute to resistance, increasing evidence suggests that non-genetic adaptations play equally critical roles.

#### 2.2.1. Non-Genetic Plasticity

Cellular plasticity is a crucial adaptive mechanism that allows cancer cells to shift between phenotypic states without developing new genomic mutations [2]. Through processes such as epithelial–mesenchymal transition (EMT), acquisition of stem cell-like properties, and lineage switching, malignant populations generate non-genetic heterogeneity that allows them to survive under intense therapeutic pressure [32,43]. These transitions are often reversible and are primarily governed by epigenetic regulation rather than by permanent changes in the DNA sequence [5,44]. This plasticity is mediated by chromatin-remodeling complexes and histone-modifying enzymes that reconfigure the transcriptional landscape. Through epigenetic modulation, cancer cells can rapidly reorganize gene expression programs in response to stress. A central feature of this adaptive process is “enhancer switching,” in which alternative enhancer elements are activated to sustain the expression of essential homeostatic regulators, including MYC, even under cytotoxic conditions [45,46]. By leveraging developmental transcriptional programs, plastic cancer cells sustain survival mechanisms while reducing vulnerabilities associated with proliferation.

**Figure 1:**
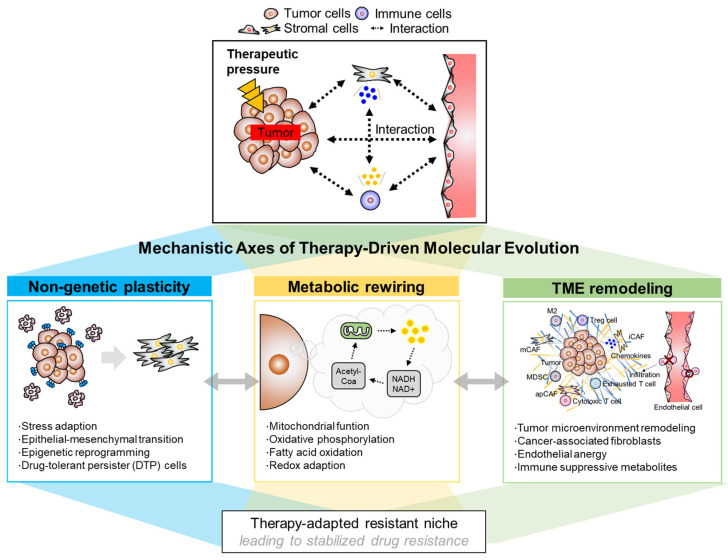
Conceptual framework of therapy-driven molecular evolution of drug resistance. Sustained therapeutic pressure drives the coordinated adaptation of tumor cells through three interconnected mechanistic axes: non-genetic plasticity, metabolic rewiring, and tumor microenvironment (TME) remodeling. Non-genetic plasticity facilitates reversible phenotypic transitions and epigenetic adaptation. Metabolic rewiring shifts tumor cells toward survival-oriented metabolic states, emphasizing mitochondrial function and redox regulation. TME remodeling creates a resistance-supportive niche through stromal activation, extracellular matrix remodeling, vascular dysfunction, and immune suppression. Through reciprocal interactions, these three axes converge to establish a survival-optimized resistant state, reflecting a growth–survival trade-off and the progressive stabilization of drug resistance.

One functional consequence of this reprogramming is the emergence of drug-tolerant persister (DTP) cells during acute therapeutic exposure. DTP cells exhibit transient resistance without stable genetic alterations, and they often display stem-like characteristics, metabolic flexibility, and increased tumor-initiating potential [29,30,31,32,33,34,47]. Importantly, the DTP state should not be viewed as a passive or terminal intermediate. Instead, it may serve as a dynamic reservoir enriched for stress-adaptive processes, including metabolic reorganization and stress-associated mutagenic activity [30,31]. In this context, transient non-genetic adaptation can create conditions that help in the eventual acquisition and stabilization of heritable resistance traits. From an evolutionary standpoint, this process acts as a bridge: reversible phenotypic adaptations occur first and may encourage the development of more stable, genetically encoded resistance phenotypes [30,33]. Consequently, the evolution of resistance cannot be fully explained by clonal selection acting solely on pre-existing genetic variants. Instead, it reflects an integrated process where reversible state transitions reshape cellular state space, alter fitness landscapes, and interact with subsequent genetic selection. Therapy-induced resistance should, therefore, be conceptualized as a multi-layered evolutionary trajectory that encompasses both genetic selection and non-genetic state-space reconfiguration. Cellular plasticity does not replace clonal evolution; rather, it expands the spectrum of selectable phenotypes on which evolutionary forces can act (Figure 1).

#### 2.2.2. Metabolic Rewiring

Metabolic plasticity represents a second major axis of therapy-induced evolution. Although proliferating cancer cells often show an increased glycolysis profile (known as the Warburg effect), tumor metabolism is neither fixed nor uniform [6]. Rather, it undergoes a flexible, context-specific, and dynamic reconfiguration during disease progression and treatment-related stress. Accumulating evidence suggests that metabolic dependencies evolve as tumors transition from treatment-naive to resistant and metastatic stages [2,7,39]. Under therapeutic pressure, cancer cells often shift from an anabolic growth program to a survival-oriented phenotype, increasing their reliance on oxidative phosphorylation (OXPHOS), fatty acid oxidation, and redox pathways [6]. This transition demonstrates an adaptation to microenvironmental constraints such as nutrient deprivation, hypoxia, and oxidative stress, rather than merely reversing the Warburg phenotype. Notably, this metabolic reconfiguration is shaped by both intrinsic genetic variations and extrinsic environmental pressures [31]. Specific mutational combinations can create novel metabolic vulnerabilities, while regional heterogeneity within tumors further diversifies metabolic states. During metastasis and the development of therapy resistance, enhanced antioxidant capacity and mitochondrial function often play critical roles in determining cell viability [48]. Beyond energy production, metabolic intermediates actively regulate signaling pathways, epigenetic states, and immune responses. Thus, metabolic reprogramming should not be seen as a mere compensatory adjustment but rather as an integrated process that orchestrates intracellular survival circuits and environmental adaptation [6]. From this perspective, therapy-induced resistance involves not only the selection of genetic clones but also the evolution of metabolic phenotypes that reshape the cellular survival landscape over time (Figure 1).

#### 2.2.3. Tumor Microenvironment Remodeling

**Figure 2:**
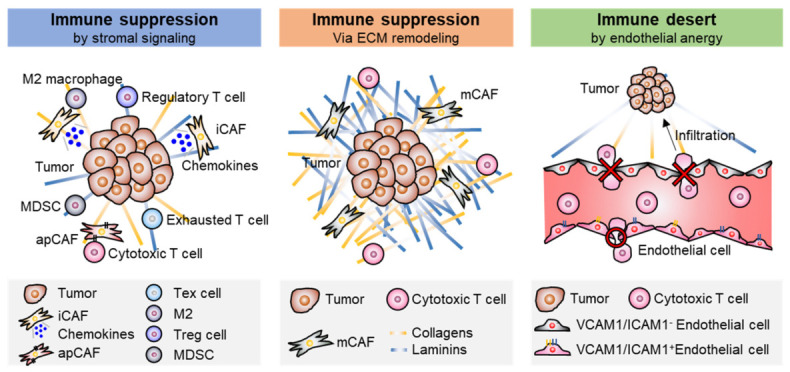
Schematic illustration of stromal and vascular mechanisms that limit anti-tumor immune infiltration. Inflammatory and antigen-presenting cancer-associated fibroblasts (iCAFs and apCAFs) promote immune dysfunction by recruiting immunosuppressive cells, including regulatory T cells, tumor-associated macrophages, and myeloid-derived suppressor cells. Myofibroblastic CAFs (mCAFs) remodel the extracellular matrix through collagen and laminin deposition, creating a physical barrier that restricts cytotoxic T-cell infiltration. Additionally, tumor-associated endothelial cells may develop a state of endothelial anergy, characterized by reduced expression of adhesion molecules such as ICAM-1 and VCAM-1, which impairs leukocyte adhesion and transendothelial migration. Together, these stromal, structural, and vascular mechanisms contribute to immune exclusion within the tumor microenvironment. The vascular components of the tumor microenvironment (TME) also undergo adaptive changes that affect immune accessibility. Tumor-associated endothelial cells frequently exhibit endothelial anergy, leading to decreased expression of adhesion molecules like ICAM-1 and VCAM-1. This phenotype hinders leukocyte adhesion and transendothelial migration, further limiting immune cell infiltration into tumor tissues [49,52,53,54]. Such vascular remodeling effectively creates an immune-excluded microenvironment that shields tumor cells from cytotoxic immune surveillance (Figure 2).

Therapy-driven evolution is not limited to the intrinsic rewiring of cancer cells; it is also significantly influenced by the dynamic remodeling of the tumor microenvironment (TME) [49]. Under therapeutic stress, tumor cells reprogram their secretory profiles, including cytokines, metabolites, extracellular vesicles (EVs), and matrix components. This reprogramming reshapes stromal architecture, vascular function, and immune composition within the tumor ecosystem [9,11,13,14,49,50]. These secretome-driven interactions establish a microenvironment that favors survival rather than eradication (Figure 2).

A central driver of this remodeling process is the activation and diversification of cancer-associated fibroblasts (CAFs). Different CAF subpopulations contribute to resistance through complementary mechanisms. Inflammatory CAFs (iCAFs) secrete high levels of chemokines and cytokines, including IL-6, CXCL12, and CCL2, which promote the recruitment of immunosuppressive cells and reinforce T-cell exhaustion within the tumor environment [11,12,14,49,50]. Myofibroblastic CAFs (mCAFs), on the other hand, primarily contribute to structural remodeling by producing extracellular matrix components such as collagen and laminin. The increased deposition of ECM and the resulting matrix stiffness can physically restrict immune cell infiltration and create spatially protected survival niches [12,14,49,50,51]. A third subset, antigen-presenting CAFs (apCAFs), has been proposed to function as noncanonical antigen-presenting cells. By presenting antigens without the necessary co-stimulatory signals, apCAFs may induce T-cell anergy and contribute to localized immune tolerance [14,49,50]. Through these diverse activities, CAF populations act as key architects of a therapy-adapted stromal landscape.

The immune compartment is reshaped under chronic therapeutic pressure. Cytokine networks involving TGF-β, IL-10, CXCL8, and CCL2 promote the expansion of immunosuppressive populations, including regulatory T cells (Tregs), tumor-associated macrophages (TAMs), and myeloid-derived suppressor cells (MDSCs). Additionally, macrophage polarization toward M2 phenotypes further reinforces immunosuppressive signaling [10,14,31,49,50,52]. In addition, chronic antigen stimulation and inhibitory signaling contribute to the emergence of exhausted T cells and NK cells, while regulatory B cells may further suppress anti-tumor immune responses [55,56,57,58,59]. Metabolic byproducts such as lactate and adenosine accumulate within the TME and amplify these immunoregulatory effects by impairing cytotoxic lymphocyte function (Figure 2). The combined effects of stromal, vascular, and immune changes turn therapy resistance into a systems-level issue. Instead of viewing therapy resistance as isolated genetic escape events, it should be understood as an ecological adaptation, where tumor cells and various stromal components co-evolve under selective pressures, resulting in spatially diverse but functionally integrated survival networks.

While these elements provide a theoretical framework for understanding therapy-driven evolution, the degree to which they converge to influence actual survival trajectories needs empirical validation. To connect these mechanistic principles with observable evolutionary patterns, it is crucial to track the adaptive journey of cancer cells in a controlled experimental setting. Accordingly, the following section employs the GRC model as a longitudinal perspective to investigate whether these theoretical pressures—ranging from phenotypic plasticity to metabolic shifts—translate into measurable evolutionary divergence in the real-time acquisition of resistance.

## 3. Dynamic Trajectory of Molecular Evolution in Previously Established Bladder Cancer GRC Models

### 3.1. Phenotypic Divergence of T24GRC and 5637GRC

The acquisition of gemcitabine resistance does not occur along a single linear trajectory. Instead, even under the same drug-induced selective pressure, cancer cells evolve by employing various adaptive strategies over time. The established T24GRC and 5637GRC models offer experimental frameworks to longitudinally trace the non-linear trajectories of gemcitabine resistance in different contexts of bladder cancer [15,16].

**Figure 3:**
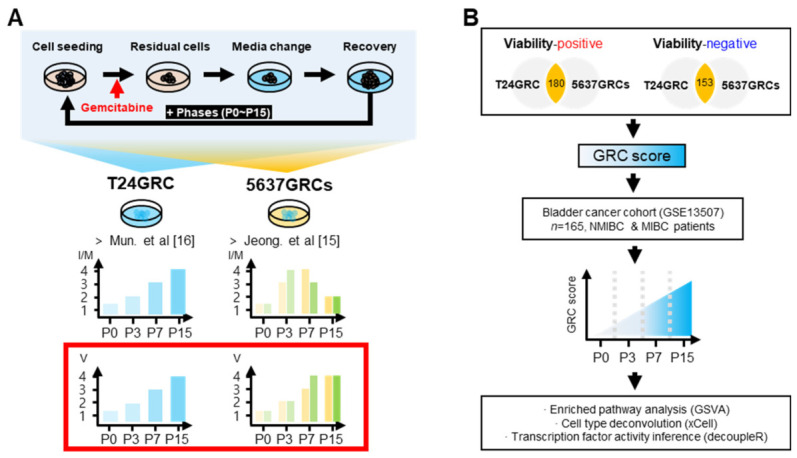
Derivation and exploratory clinical projection of the GRC score based on previously published gemcitabine-resistance models. (**A**) Gemcitabine-resistant cell (GRC) models were generated through stepwise drug exposure and recovery cycles, producing sequential phases (P0–P15) representing progressive adaptation to therapeutic stress. In the present review, directionally consistent transcriptional changes across these previously published GRC systems were integrated to derive an exploratory GRC score. The red box in panel A highlights the viability (V) readouts used to define viability-associated gene sets for GRC-score construction. (**B**) Overlapping positively and negatively correlated gene sets were used to construct the GRC score. The score was subsequently applied to a bladder cancer cohort (GSE13507; *n* = 165, including NMIBC and MIBC) to evaluate transcriptomic patterns associated with resistance evolution, followed by pathway enrichment (GSVA), cell-type deconvolution (xCell), and transcription factor activity inference (decoupleR). Data sources: T24GRC model, Mun et al. [16]; 5637GRC model, Jeong et al. [15]; bladder cancer cohort, GSE13507 [60].

In the T24GRC lineage, cumulative gemcitabine exposure leads to a stepwise and relatively synchronized increase in both invasion/migration (I/M) capacity and cell viability (V). This pattern exemplifies a concordant adaptation model, where enhanced invasiveness and survival–tolerance evolve in parallel and reinforce each other. As resistance progresses from P0 (drug-sensitive) to P15 (fully resistant), both phenotypic axes move in the same direction, indicating coordinated network remodeling that supports aggressive and drug-tolerant growth (Figure 3A) [16].

In contrast, the 5637GRC lineage follows a distinct evolutionary path. Although cell viability steadily increases across passages, the invasive/mesenchymal (I/M) phenotype exhibits a non-linear trajectory, peaking at the intermediate P7 stage and then declining at the late P15 stage. This divergence suggests that resistance evolution can differentially exploit the invasiveness axis and the survival–tolerance axis over time. Notably, the transient I/M peak observed at P7 in 5637GRC may correspond to a hybrid epithelial–mesenchymal transition (EMT) state or a maximally plastic transcriptional state. This transient plastic state is likely selected under acute gemcitabine stress but may later stabilize into a more fixed resistant phenotype or partially revert as long-term survival strategies take precedence. (Figure 3A) [15].

These findings collectively suggest that the acquisition of resistance is not a straightforward cumulative process. Rather, it follows a cyclic dynamic of plasticity and stabilization, where transient adaptive states are selected, refined, and ultimately consolidated into stable resistant phenotypes (Table 1).

### 3.2. Definition of the GRC Score

To quantitatively model the trajectory of resistance acquisition, we developed the GRC score, an integrative metric that reflects the molecular evolution from P0 (drug-sensitive) to P15 (fully resistant) states in T24 and 5637 cells exposed to stepwise gemcitabine treatment. The GRC score was constructed from genes that exhibited consistent directional changes throughout resistance evolution. Genes upregulated in resistant states and those downregulated in resistant states were organized into directional signature sets, and GSVA-based enrichment was employed to quantify how closely each clinical sample recapitulated the resistant trajectory. No additional gene-specific weighting was applied. Furthermore, the GRC score interprets resistance not merely as a simple upregulation of specific genes but as a trajectory shift within cellular state space (Figure 3B). This framework posits that resistance evolution represents a gradual and cumulative network reprogramming process. Instead of concentrating on isolated molecular alterations, the GRC score captures coordinated transcriptional changes that define progressive adaptation.

To assess its clinical relevance, we projected the GRC signature onto a bladder cancer clinical cohort (GSE13507, n = 165) [60] and performed Gene Set Variation Analysis (GSVA). Patients with high GRC scores demonstrated systematic alterations in pathways related to cell-cycle suppression, enhanced metabolic flexibility, strengthened stress-response programs, and activation of microenvironment-related signaling pathways. These findings suggest that certain transcriptomic features linked to resistance evolution in the GRC models may also be observed in clinical bladder cancer cohorts, although this should be viewed as exploratory rather than direct clinical validation. Additionally, correlation analyses between the GRC score and key biological pathways indicate that resistant states are not characterized by increased proliferation. Instead, they represent a strategic balance between growth and survival, where proliferative capacity is reduced while survival maximization programs are strengthened. This highlights the need to view chemoresistance not as a fixed endpoint but as an adaptive evolutionary strategy within dynamic molecular networks (Figure 3B). Overall, the GRC framework and score offer both a conceptual and quantitative basis for understanding chemoresistance as a temporally structured process of state-space reorganization driven by plasticity, metabolic rewiring, and microenvironmental adaptation.

### 3.3. Overview of Molecular Evolution for Drug Resistance: The Growth–Survival Trade-Off (P0→P15)

**Figure 4:**
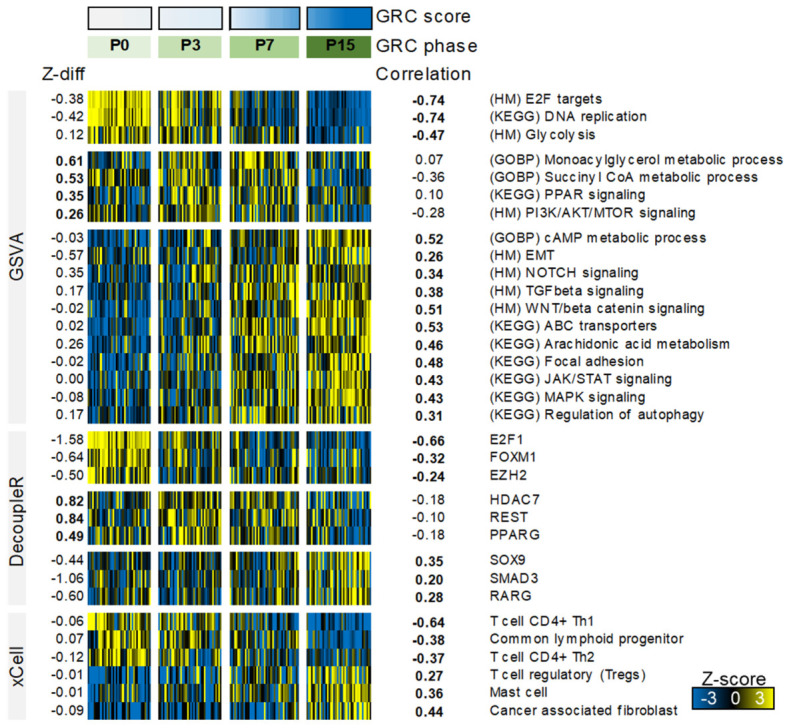
Integrated heatmap of GRC score-associated molecular features across resistance evolution. Heatmaps summarize GSVA pathway activity, DecoupleR-inferred transcription factor activity, and xCell-derived cell-type signatures across GRC phases (P0, P3, P7, and P15). Rows are grouped by analytical method, and columns represent samples ordered by increasing GRC score. Left-side Z-diff values indicate phase-enriched features, whereas right-side coefficients indicate correlations with the GRC score in the clinical cohort. Z-diff refers to the difference in Z-value between P3 and P7 and the remaining stages. The figure highlights a shift from proliferation-associated programs in P0 toward stress-adaptive signaling, late-stage transcriptional regulators, and stromal/immunoregulatory microenvironmental features as resistance progresses. Data sources: bladder cancer cohort, GSE13507 [60].

GSVA analysis of the GSE13507 cohort revealed a strong negative correlation between the GRC score and the primary proliferation pathways of cancer cells, alongside a robust positive correlation with pathways related to survival and environmental adaptation. This suggests that as resistance strengthens, cancer cells transition from a state of rapid division to a low-energy survival mode (Figure 4).

#### 3.3.1. Sacrificing Growth for Survival

The correlation analysis revealed a strong inverse association between the GRC score and E2F target pathway activity (R = −0.74), which is a transcriptional program central to cell-cycle progression and S-phase entry (Table 2). This pattern suggests that tumors with higher GRC scores tend to display reduced proliferation-related transcriptional signatures [2]. Since gemcitabine primarily exerts its cytotoxic effect by interfering with DNA synthesis during the S-phase, the observed downregulation of E2F-driven programs aligns with a cellular state marked by reduced cell-cycle activity [29,48]. Instead of demonstrating a direct causal blockade of S-phase entry, these findings suggest that resistant states are transcriptionally linked to a reduced proliferative drive [6,7]. This inverse relationship supports a model in which high-GRC tumors exist in a molecular state that is less influenced by rapid cell-cycle progression. This state may decrease their vulnerability to S-phase-dependent cytotoxic stress.

#### 3.3.2. Energy-Saving Mode: Metabolic Downshift and Defense Reinforcement

Cancer cells are commonly characterized by elevated glycolytic activity that supports rapid proliferation. In this analysis, glycolysis-related pathways exhibited a negative correlation with the GRC score (R = −0.47), while mTORC1 signaling, a central regulator of anabolic metabolism, showed an even stronger inverse association (R = −0.68). These findings suggest that tumors with higher GRC scores tend to display reduced transcriptional signatures associated with growth-related metabolic programs [13,29]. Pathways related to cellular defense and stress adaptation showed positive correlations with the GRC score. Notably, ABC transporter pathways (R = 0.53) and autophagy-related programs (R = 0.31) were enriched in high-GRC tumors (Table 2). This reciprocal pattern indicates a transcriptional shift where anabolic and proliferation-related metabolic programs are diminished, while stress-response and survival-associated programs become more prominent [6,9,29]. Rather than suggesting a complete metabolic reprogramming or a definitive switch to specific energy sources, these data support a model of metabolic reallocation at the transcriptional level. High-GRC tumors seem less dominated by biosynthetic and cell cycle-related metabolic pathways and are more associated with programs involved in damage tolerance, substrate recycling, and cellular defense. This pattern is consistent with a growth–survival trade-off, in which reduced anabolic signaling is coupled with the reinforcement of stress-adaptive mechanisms.

#### 3.3.3. Remodeling the Tumor Microenvironment (TME)

In addition to cell-intrinsic adaptations, the GRC score was associated with distinct alterations in the tumor microenvironment (TME). Correlation analysis using xCell-derived cell-type signatures revealed that tumors with higher GRC scores showed a significant reduction in Th1 CD4^+^ T-cell signatures (R = −0.64), which are typically associated with effective anti-tumor immune responses [11,52]. In contrast, signatures associated with regulatory T cells (Tregs) (R = 0.27) and cancer-associated fibroblasts (CAFs) (R = 0.44) showed positive correlations with the GRC score (Table 2) [12,14,25,27]. These reciprocal patterns indicate that tumors with higher GRC scores are characterized by reduced pro-inflammatory immune activity and increased stromal-related signals.

This interpretation is supported by recent experimental and clinical studies showing that CAFs can actively generate resistance-supportive niches rather than merely reflecting passive stromal expansion. In bladder cancer, SLC14A1^+^ CAFs have been shown to promote cancer stemness through WNT5A signaling, while CAF-derived exosomal miR-146a-5p enhances urothelial cancer stemness and resistance to gemcitabine and cisplatin [61,62]. Similar CAF-mediated immune exclusion mechanisms have been reported beyond bladder cancer. For instance, TGF-β-dependent LRRC15^+^ myofibroblasts suppress CD8^+^ T-cell function and limit responsiveness to immune checkpoint blockade [63]. These studies support the notion that CAF-rich or ECM-remodeled tumors create both biochemical and physical barriers to effective anti-tumor immunity. Moreover, spatial profiling studies in muscle-invasive bladder cancer indicate that the spatial organization of tumor and immune compartments can enhance the prediction of pathological response to neoadjuvant chemoimmunotherapy [64]. This stromal- and Treg-enriched landscape resembles the immune-excluded phenotype observed in several solid tumors, where physical and biochemical barriers restrict effective immune infiltration [29,34,52]. Within the framework of resistance evolution, such a microenvironmental configuration may contribute to the stabilization of drug-tolerant cellular states.

### 3.4. Molecular Evolution for Drug Resistance: Metabolic Reorganization and Acquisition of Non-Genetic Plasticity (P3→P7)

Acquiring drug resistance involves more than just enhancing cell viability; it requires a fundamental reallocation of energy resources. The GSVA results from our GRC model reveal a dual molecular framework in the resistance process: a reduction in proliferation-focused metabolic programs paired with a selective enhancement of metabolic pathways that support survival. Notably, the decrease in glycolysis, along with the activation of lipid metabolism and mitochondrial pathways during the P3/P7 stages, clearly illustrates that the evolution of resistance adheres to a “growth–survival trade-off” strategy.

#### 3.4.1. Lipid-Associated Transcriptional Enrichment During the Intermediate Phase

During the P3–P7 stages, gene sets related to the monoacylglycerol metabolic process (Z-diff = 0.60, 7/481) and triglyceride metabolic processes, along with the PPAR signaling pathway (Z-diff = 0.34, 13/186), showed relative enrichment compared to earlier passages (Table 3). These findings suggest that intermediate-stage resistant cells exhibit an increased transcriptional representation of lipid-associated metabolic programs. The PPAR family of nuclear receptors is widely recognized as regulators of genes involved in fatty acid metabolism, mitochondrial function, and cellular stress adaptation [7]. Accordingly, the enrichment of PPAR-related signatures in the P3/P7 window suggests that lipid-linked regulatory networks become more prominent during this transitional phase. Rather than indicating a definitive metabolic switch from glucose dependence to lipid utilization, the current data highlight a redistribution of metabolic transcriptional emphasis, where lipid-associated programs gain relative importance within the overall metabolic landscape [9]. This pattern may indicate an adaptive rebalancing under prolonged drug exposure, where biosynthetic and proliferation-centered pathways are comparatively diminished, while stress-associated metabolic programs become more pronounced [6,48]. However, direct assessment of metabolic flux is necessary to determine whether these transcriptional shifts result in altered substrate utilization. Within the current framework, the enrichment of lipid-related pathways is best interpreted as a marker of metabolic reorganization that occurs during the intermediate, plastic phase of resistance evolution.

#### 3.4.2. Enrichment of Succinyl-CoA-Associated Pathways and TCA-Linked Programs

The intermediate resistance phase (P3–P7) was characterized by a relative enrichment of gene sets associated with the succinyl-CoA metabolic process (Z-diff = 0.53, 12/481, Table 3). Since succinyl-CoA is a central intermediate in the tricarboxylic acid (TCA) cycle, this enrichment suggests an increased transcriptional representation of TCA-linked metabolic programs during this stage [7,48]. Rather than showing a clear increase in mitochondrial respiration or oxidative phosphorylation, these findings suggest that genes associated with mitochondrial metabolic processes become more prominent in the broader transcriptional landscape of intermediate-stage cells [48]. Such enrichment aligns with a rebalancing of metabolic networks, where TCA-associated pathways gain relative importance during the early adaptation to sustained drug exposure. In addition to its role in energy production, the TCA-cycle metabolism also interacts with cellular signaling and epigenetic regulation [5,13]. Metabolites like succinate have been linked to chromatin-associated modifications, such as protein succinylation, as well as hypoxia-related signaling pathways [6].

While the current data do not directly assess metabolite levels or signaling activity, the observed transcriptional enrichment suggests that metabolic reorganization during the P3–P7 window may intersect with regulatory programs beyond energy production. Combined with the lipid-associated pathway enrichment mentioned earlier, the relative increase in TCA-linked gene sets supports the idea that the intermediate phase of resistance is characterized by a coordinated transcriptional reconfiguration of mitochondrial-related metabolic programs.

#### 3.4.3. Acquisition of Epigenetic Plasticity

The intermediate adaptation stages (P3 and P7) were characterized by an increase in the inferred regulatory activity of several epigenetic regulators, including REST (Z-diff = 0.83), HDAC7 (Z-diff = 0.80), and MECP2 (Z-diff = 0.69; Table 3). These factors are broadly implicated in chromatin remodeling and transcriptional regulation, and their relative enrichment during this period suggests enhanced engagement of epigenetic regulatory programs. Rather than indicating a definitive reprogramming event, the elevated representation of these regulators supports the notion that the P3–P7 interval is associated with increased transcriptional flexibility [2,5,34,42,43]. Epigenetic regulators such as REST, HDAC7, and MECP2 modulate chromatin accessibility and gene expression dynamics, processes that can enable reversible phenotypic transitions in response to environmental stress [2,5]. In the context of stepwise gemcitabine exposure, the enrichment of epigenetic regulatory signatures during the intermediate phase suggests a transiently plastic state in which transcriptional programs are more dynamically modulated. This interpretation aligns with the broader pattern observed in passages P3 to P7, where metabolic and regulatory networks seem to undergo coordinated rebalancing before stabilizing at later passages.

### 3.5. Molecular Evolution for Drug Resistance: Interaction with the Tumor Microenvironment (TME) via Signaling Pathways (P7→P15)

Correlation analysis between the GRC score and various signaling pathways indicates that resistance evolution goes beyond internal transcriptional and metabolic rewiring; it actively reconstructs interactions with the tumor microenvironment (TME). Specifically, signaling networks such as the PI3K/AKT, MAPK, WNT, NOTCH, and ECM-adhesion pathways function as hubs that integrate metabolic status, survival signals, and cell-fate decisions to create a protective niche.

#### 3.5.1. PI3K/AKT and MAPK Signaling Pathways

The PI3K/AKT and MAPK pathways are widely recognized as central signaling networks integrating extracellular cues with intracellular growth and survival responses [4,21,50]. In the present analysis, MAPK signaling showed a positive correlation with the GRC score (R = 0.43), indicating that high-GRC tumors have increased transcriptional representation of MAPK-associated gene sets (Table 4). Similarly, PI3K/AKT/mTOR-related signatures exhibited modest enrichment during the intermediate and late stages (Z-diff = 0.25, Table 3). These observations suggest that resistant states are associated with the relative preservation or enhancement of signaling programs linked to growth factor response and stress adaptation [21,50,53]. However, the current data reflect pathway-level transcriptional enrichment rather than direct measurements of kinase activity or phosphorylation status. Therefore, the findings should be interpreted as evidence that MAPK and PI3K/AKT-related transcriptional programs remain prominent in resistant contexts. Within the broader growth–survival trade-off framework described above, the persistence of these signaling signatures, alongside reduced anabolic and glycolytic programs, may indicate a rebalancing of proliferative and stress-responsive networks.

#### 3.5.2. WNT and NOTCH Signaling Pathways

The WNT/β-catenin and NOTCH signaling pathways are widely recognized regulators of cell–cell fate determination and developmental processes and have been frequently implicated in cancer progression and cellular plasticity [1,43]. In this analysis, WNT signaling exhibited a positive correlation with the GRC score (R = 0.51), while NOTCH signaling displayed a moderate positive association (R = 0.34). This indicates an increased transcriptional representation of these pathway-associated gene sets in high-GRC tumors (Table 4). The enrichment of WNT-related signatures suggests that β-catenin-associated transcriptional programs become more pronounced in resistant contexts. Previous studies have linked these programs to stemness-related phenotypes, drug tolerance, and interactions with epithelial–mesenchymal transition (EMT) pathways [1,2,34,40]. Similarly, the relative enrichment of NOTCH-associated gene sets may indicate an increased engagement of signaling networks involved in cell-state transitions and regulatory plasticity. Within the broader context of resistance evolution, the coordinated representation of WNT and NOTCH signatures is consistent and aligns with a model in which developmental signaling pathways contribute to the stabilization of adaptive cellular states [1,43].

#### 3.5.3. ECM Remodeling and Hippo–YAP/TAZ Signaling Pathway

Extracellular matrix (ECM) organization and cell–matrix interactions are key determinants of tumor architecture and signaling context [12,49,50]. In this analysis, gene sets associated with Focal Adhesion (R = 0.48) and ECM–receptor interaction (R = 0.34) showed positive correlations with the GRC score. This indicates a greater transcriptional representation of ECM-related programs in high-GRC tumors (Table 4). The enrichment of these signatures indicates that resistant states are linked to an enhanced representation of cell–matrix interaction pathways at the transcriptional level. Prior studies have shown that ECM-associated signaling interacts with mechanotransduction pathways, including the Hippo–YAP/TAZ axis [41,49,50]. Accordingly, the observed pattern suggests that ECM-related transcriptional programs and Hippo/YAP-linked gene sets may become more prominent during the later stages of resistance evolution. Within the broader context of resistance stabilization, the coordinated enrichment of ECM-related and mechanotransduction-associated gene sets supports a model in which cell–matrix signaling programs are transcriptionally linked to resistant phenotypes [43].

#### 3.5.4. VEGF Signaling Pathway

VEGF-related gene sets showed a modest positive correlation with the GRC score (R = 0.23), indicating that VEGF-associated signaling is more transcriptionally represented in high-GRC tumors (Table 4). While the strength of this association is moderate, it suggests that angiogenesis-related transcriptional programs may become more prominent as resistance progresses. VEGF signaling is widely recognized for its role in vascular regulation and tumor-associated angiogenesis. Additionally, previous studies have linked it to hypoxia-responsive pathways and growth factor signaling networks, such as PI3K/AKT and MAPK [4,49,50]. In this context, the observed enrichment of VEGF-associated signatures suggests that vascular and hypoxia-related transcriptional programs may contribute to a broader resistant state [20]. In conjunction with the metabolic and signaling reconfiguration discussed in earlier sections, the relative increase in VEGF-related transcriptional programs suggests a model where resistance-associated states are linked to a greater representation of microenvironment-responsive signaling networks.

#### 3.5.5. EMT and Axon Guidance

Gene sets related to Axon Guidance showed a positive correlation with the GRC score (R = 0.39), indicating an increased transcriptional representation of this pathway in resistant contexts (Table 4). The Axon Guidance category encompasses various ligand–receptor systems, including semaphorins (SEMAs) and ephrin family members (EFNAs), which are traditionally involved in neuronal migration and boundary formation. In cancer biology, these signaling systems have been linked to processes such as cell migration, tumor–stroma interactions, and microenvironmental organization [65,66,67,68]. Accordingly, the observed enrichment of Axon Guidance-associated signatures suggests that ligand–receptor-mediated communication networks may become more prominent during the later stages of resistance evolution. In particular, prior studies have linked Eph/ephrin signaling to contact-dependent signaling and spatial organization within tumors. Within the broader framework of resistance, the coordinated enrichment of EMT- and Axon Guidance-associated gene sets aligns with a model in which cell-state plasticity and intercellular signaling networks are integrated during the stabilization of resistant phenotypes [2,49,50].

### 3.6. Molecular Evolution for Drug Resistance: Interaction with the Tumor Microenvironment (TME) via Secreted Factors (P7→P15)

Chemotherapy exerts a powerful selective pressure that goes beyond mere cell death, reshaping the entire tumor ecosystem. This pressure reorganizes extracellular signaling networks, metabolic balance, and redox homeostasis, ultimately creating a microenvironment marked by the accumulation of specific secreted metabolites and cytokines. This remodeling of the secretome fosters a localized survival niche that enables the selection and expansion of resistant cells.

#### 3.6.1. Secreted Factors-Associated Signatures and Paracrine Signaling

Gene sets associated with secreted factors and extracellular signaling molecules were enriched in high GRC states. In particular, focal adhesion pathways related to cell adhesion (R = 0.48) exhibited a strong positive correlation with GRC scores (Table 4). Genes contributing to these pathways include several components related to the extracellular matrix, such as collagen family members (COLs), laminin subunits (LAMAs), and caveolin proteins (CAVs) [51,69,70,71,72]. These molecules are essential structural and signaling components of the extracellular matrix and the cell–matrix adhesion system. Collagens and laminins contribute to the formation of the extracellular skeleton and basement membrane structures, while caveolins are involved in membrane-associated signaling and mechanical signaling processes. The enrichment of these extracellular matrix-related factors suggests that the resistant state may be linked to transcriptional programs that enhance cell–matrix interactions and communication with the microenvironment. In addition to conventional ECM remodeling, emerging evidence indicates that resistant tumors may also utilize atypical glyco-immunoregulatory mediators. Cancer-cell-derived immunoglobulin G-related molecules, particularly sialylated cancer IgG (SIA-IgG), have been identified in several epithelial cancers. These molecules may function less as conventional antigen-specific antibodies and more as noncanonical signaling glycoproteins. Through interactions with Siglec receptors on T cells or macrophages, SIA-IgG may reduce cytotoxic activity and promote immunosuppressive polarization. However, this mechanism remains only partially validated, and bulk immunoglobulin signals must be carefully distinguished from B-cell or plasma-cell infiltration [73,74,75].

#### 3.6.2. Adenosine and cAMP-Related Signaling

Among the metabolic pathways associated with the GRC score, gene sets related to the cyclic AMP (cAMP) biosynthetic process exhibited a strong positive correlation (R = 0.59). Additionally, genes involved in the adenosine metabolic process also showed a positive correlation with the GRC score (R = 0.30), indicating an increased transcriptional representation of nucleotide-associated signaling pathways in high-GRC tumors (Table 5). cAMP serves as a central second messenger that integrates extracellular stimuli with intracellular signaling programs involved in cellular stress responses and metabolic regulation. In the tumor microenvironment, cAMP signaling is closely linked to purinergic signaling networks, where extracellular nucleotides and their metabolites act as mediators of intercellular communication [76,77]. A key component of this axis involves the enzymatic conversion of extracellular ATP into adenosine via the ectonucleotidases CD39 and CD73, a process that generates adenosine-rich microenvironments capable of activating purinergic receptors [76,77,78,79,80,81,82]. Activation of these receptors stimulates adenylate cyclase, elevating intracellular cAMP levels and linking extracellular nucleotide metabolism with intracellular signaling responses. In this context, the concurrent enrichment of adenosine metabolic programs and cAMP-associated signaling pathways suggests that adenosine–cAMP signaling axes may play a role in the broader signaling landscape associated with resistance evolution, potentially enhancing adaptive cell–cell communication and stress tolerance within the tumor microenvironment.

#### 3.6.3. Arachidonic Acid

Arachidonic acid metabolism-related gene sets were positively correlated with the GRC score (R = 0.30, Table 5), indicating an increased transcriptional representation of lipid-derived inflammatory signaling pathways in resistant tumors. Arachidonic acid acts as a central substrate for the production of eicosanoids, such as prostaglandins and leukotrienes, through the cyclooxygenase (COX) and lipoxygenase (LOX) enzymatic pathways [30,83]. These lipid mediators are extensively involved in regulating tumor-associated inflammation and immune modulation within the tumor microenvironment. In particular, prostaglandin E2 (PGE2), a key downstream product of arachidonic acid metabolism, has been shown to affect various components of the tumor ecosystem. This includes the suppression of cytotoxic T-cell activity, the recruitment of immunosuppressive myeloid populations, and the modulation of stromal cell function [84,85]. In addition to their role in immune regulation, arachidonic acid-derived signaling molecules are also linked to angiogenic processes and the regulation of vascular permeability. This connection ties inflammatory signaling to vascular remodeling in tumors. The observed enrichment of arachidonic acid-associated gene sets indicates that lipid-derived inflammatory signaling networks may become more transcriptionally prominent in resistant tumor states. Within the larger context of resistance evolution, these pathways may help establish a microenvironment that supports immune evasion, stromal remodeling, and adaptive tumor survival.

#### 3.6.4. Role of Lactate, ROS, and Redox Re-Equilibration

Lactate metabolism and reactive oxygen species (ROS) signaling play significant roles in shaping metabolic and immune dynamics within the tumor microenvironment. Lactate, a primary byproduct of glycolytic metabolism, is increasingly recognized not just as a metabolic substrate but also as a signaling molecule that influences the behavior of stromal and immune cells. The accumulation of extracellular lactate in tumors has been shown to modulate immune cell activity, including the suppression of cytotoxic T-cell and natural killer cell functions, while promoting the differentiation of immunosuppressive cell populations. Concurrently, alterations in ROS production and redox homeostasis are often observed in therapy-resistant cancer cells, indicating adaptations in oxidative stress management and mitochondrial metabolism [7,86,87,88,89,90]. Redox regulatory systems can influence multiple signaling pathways and transcriptional programs related to survival under therapeutic stress. In this context, the transcriptional enrichment of redox- and metabolism-associated gene sets in high-GRC states aligns with previous observations that resistant tumors often show altered oxidative stress management programs.

## 4. Therapeutic Implications for Controlling the Evolution of Resistance

The evolutionary trajectory outlined by the GRC framework suggests that therapeutic interventions may be more effective when they align with the temporal dynamics of resistance acquisition rather than focusing solely on fully established resistant clones. In this context, the intermediate adaptation window (P3–P7) represents a therapeutically significant transitional state in which resistance is not yet fully stabilized. The molecular features observed during this phase—including the activation of focal adhesion, PI3K–AKT, TGF-β, and chromatin-remodeling programs—support the rationale for targeting adaptive plasticity before resistant phenotypes become fixed. Consequently, inhibitors of FAK, AKT, NOTCH, YAP1, and TGF-β signaling, as well as epigenetic modulators that disrupt reversible persister-like states, may be particularly relevant during this interval [91,92,93,94,95].

More specifically, non-genetic plasticity may be therapeutically addressed through modulation of chromatin-dependent state transitions. Inhibitors of DNMT, HDAC, BET, LSD1, and EZH2 have been investigated as strategies to alter lineage plasticity, transcriptional memory, antigen presentation, and immune responsiveness. In urothelial carcinoma, a phase I/II trial evaluated the EZH2 inhibitor tazemetostat in combination with pembrolizumab for locally advanced or metastatic disease, providing a clinically relevant example of an epigenetic-immunotherapy combination (NCT03854474). Additionally, the NCT03765229 study assessed entinostat-based epigenetic priming combined with pembrolizumab in patients with non-inflamed stage III/IV melanoma, representing a non-urothelial example of HDAC inhibition combined with immune checkpoint blockade to modulate tumor immune responsiveness. Metabolic reprogramming offers a second therapeutic axis. The GRC trajectory suggests that resistant states may develop context-dependent dependencies on mitochondrial metabolism, lipid-associated inflammatory signaling, redox adaptation, or mTOR-related survival programs. Therefore, OXPHOS inhibitors (NCT03291938), mTOR pathway inhibitors, AMPK-modulating agents, and lipid metabolism-directed interventions may be considered candidate strategies for targeting metabolically adapted resistant states. For instance, the mitochondrial complex I inhibitor IACS-010759 has been evaluated in phase I trials for advanced cancers, and metformin plus simvastatin (NCT02360618) has been tested as a neoadjuvant window-of-opportunity metabolic intervention in bladder cancer.

In contrast, late-stage resistance (P15) seems to rely less on transient cellular plasticity and is more closely linked to microenvironmental stabilization. The enrichment of ECM–mechanical transduction pathways, purinergic signaling, and inflammatory lipid mediator programs suggests a tumor state sustained by stromal support and the formation of a resistance-permissive niche. In this context, strategies aimed at disrupting tumor–stroma interactions, adenosine/cAMP-related signaling, or lipid-associated inflammatory circuits may be more effective in destabilizing the resistant ecosystem than targeting tumor cells alone [96,97,98,99]. Beyond conventional immune checkpoint blockade, more direct TME-modulating strategies include targeting TGF-β-mediated immune exclusion, adenosine-related immunosuppression, CAF-associated signals, and ECM remodeling. For instance, selective TGF-β1 inhibition combined with PD-1 blockade, exemplified by linavonkibart plus pembrolizumab, offers a clinically tested strategy for reversing TGF-β-associated immune exclusion (NCT04291079). Similarly, CD73 blockade with oleclumab plus durvalumab represents an adenosine-axis-directed approach to modulating immunosuppressive purinergic signaling (NCT03773666). CAF- or stromal-directed approaches, including FAP-targeted radioligand therapy such as [^177^Lu]Lu-FAP-2286, further illustrate attempts to therapeutically target the stromal compartment (NCT04939610). These representative therapeutic strategies and clinical examples targeting resistance-associated processes are summarized in Table 6.

These observations support a phase-aware therapeutic model. Instead of applying treatment as a static and uniform selective pressure, strategies informed by time-sensitive biomarkers, such as longitudinal changes in the GRC score, may help steer tumor evolution away from stabilized resistant states. From this viewpoint, the therapeutic goal shifts from merely eradicating resistant clones to actively interrupting or redirecting the evolutionary process that leads to resistance.

## 5. Research Perspectives

A critical next step is to determine whether the temporally ordered adaptive states proposed in the GRC framework can be directly observed in patient tumors. Specifically, longitudinal sampling strategies combined with single-cell transcriptomic and epigenomic profiling may clarify whether intermediate plastic states, akin to the P3–P7 window, emerge during treatment and subsequently stabilize into more durable resistant phenotypes. Recent longitudinal multi-omics studies have demonstrated that therapy can reshape both tumor cell states and the immune-stromal microenvironment over time, supporting the feasibility of this approach [100]. Equally important, future studies should clarify the spatial organization of resistance-supportive niches. The current framework implicates extracellular matrix remodeling, stromal support, vascular adaptation, and immunometabolic signaling. Therefore, spatial transcriptomics and related spatial multi-omics platforms may be particularly valuable for identifying where resistant cell states emerge and how they interact with cancer-associated fibroblasts, endothelial cells, macrophages, and exhausted lymphocytes in situ. Recent studies have shown that integrated single-cell and spatial approaches can reveal treatment-associated epithelial–myeloid interactions, immune exhaustion, and microenvironmental niches that are not detectable in bulk datasets alone [101,102]. Integrating these technologies with perturbation-based validation in organoid, co-culture, or microfluidic systems will be essential for distinguishing causal drivers of resistance from the secondary consequences of tumor evolution.

## 6. Conclusions

Drug resistance in cancer should be viewed not as a singular genetic event but as a dynamic evolutionary process influenced by ongoing therapeutic pressure. In this review, we propose a conceptual framework in which resistance develops through coordinated molecular evolution across three interconnected axes: non-genetic plasticity, metabolic reorganization, and tumor microenvironment remodeling. Using the GRC model as a temporal reference, we illustrate how cancer cells gradually shift from proliferation-dominant states to survival-optimized states, navigating a growth–survival trade-off.

**Figure 5:**
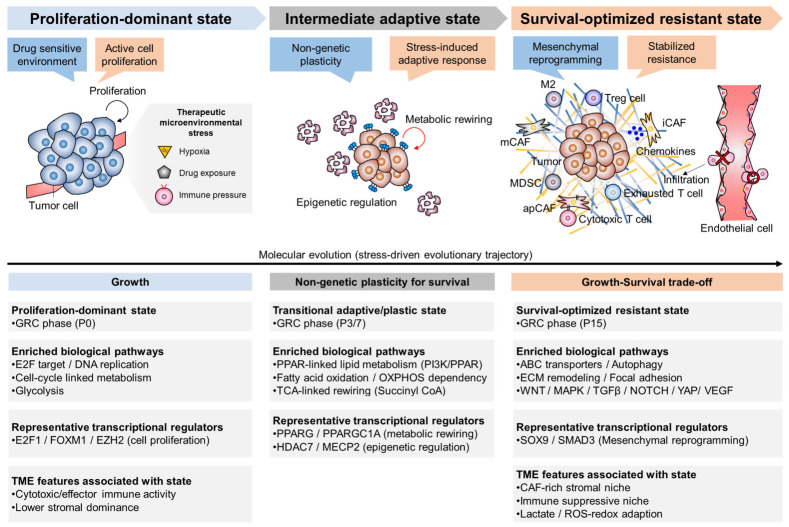
Therapy-driven evolutionary transition toward stabilized drug resistance. The schematic summarizes the progressive transition of tumor cells under therapeutic stress within the tumor microenvironment. In the proliferation-dominant state, tumors exhibit active cell-cycle programs, glycolytic metabolism, and immune-permissive conditions. Exposure to therapeutic microenvironmental stress (hypoxia, drug exposure, and immune pressure) induces a transitional adaptive state characterized by non-genetic plasticity and metabolic rewiring, including lipid metabolism and OXPHOS dependency. Through growth–survival trade-offs, tumors ultimately evolve into a survival-optimized resistant state marked by stromal remodeling, immune suppression, and the activation of resistance-associated signaling pathways. The model illustrates the coordinated interaction between cellular programs and microenvironmental remodeling during resistance evolution.

The early adaptive phases are marked by metabolic reallocation and epigenetic plasticity, while later stages involve significant interactions with the tumor microenvironment through various signaling pathways and secreted metabolites, such as adenosine and lipid-associated mediators. Additionally, projecting the GRC score onto clinical transcriptomic data suggests that these evolutionary patterns may also be mirrored in patient tumors (Figure 5).

However, several limitations should be acknowledged. First, the observed associations are primarily based on transcriptomic and correlation analyses. It is important to note that correlation does not imply causation; thus, the relationships identified between the GRC score and specific pathways or microenvironmental signatures should not be viewed as direct mechanistic drivers of resistance. Second, transcriptomic signatures do not directly reflect functional phenotypes such as proliferation rate, invasive capacity, drug tolerance, or immune evasion. Third, the current framework is mainly based on gemcitabine-resistant bladder cancer models and should not be interpreted as universally applicable across all drug classes or cancer types. While the GRC model serves as a valuable longitudinal reference system for understanding therapy-driven resistance evolution, further validation in diverse therapeutic contexts, treatment modalities, tumor types, and functional experimental systems is essential to establish the broader generalizability of the proposed growth–survival trade-off and evolutionary trajectory. Despite these limitations, conceptualizing chemoresistance as a temporally structured evolutionary process offers valuable insights into the emergence of adaptive tumor states and the development of therapeutic strategies aimed at interrupting resistance before it becomes entrenched.

## Figures and Tables

**Table 1:** Evolutionary phases of the GRC model.

Phase	T24GRC ^a^	5637GRC ^b^	Molecular State
Drug-sensitive (P0)	Drug-sensitive	Drug-sensitive	Active proliferation
Early phase (P3)	Initial plasticity	Initial plasticity	Stress-induced adaptive response
Intermediate phase (P7)	Increased motility Increased viability	Peak motility Increased viability	Metabolic reorganization Alternative pathway activation
Late phase (P15)	Peak motility Peak viability	Decrease motility Peak viability	Stabilized resistance Niche construction

^a^ T24 cell line-driven gemcitabine-resistant cancer model. ^b^ 5637 cell line-driven gemcitabine-resistant cancer model; GRC, gemcitabine-resistant cell.

**Table 2:** Correlation analysis between GRC score and biological features, including pathways, transcription factors, and the tumor microenvironment.

Methods	Biological Pathways	Correlation (r) *	Active Phase
Pathway ^a^	(HM) E2F targets	Negative (−0.74)	P0
Pathway ^a^	(KEGG) DNA replication	Negative (−0.74)	P0
Pathway ^a^	(HM) Glycolysis	Negative (−0.47)	P0
TF ^b^	E2F1	Negative (−0.66)	P0
TF ^b^	FOXM1	Negative (−0.32)	P0
TF ^b^	EZH2	Negative (−0.24)	P0
TME ^c^	Th1 CD4+ T cell	Negative (−0.64)	P0
TME ^c^	Common lymphoid progenitor	Negative (−0.38)	P0
TME ^c^	Th2 CD4+ T cell	Negative (−0.37)	P0
Pathway	(KEGG) ABC transporters	Positive (0.53)	P3, P7, and P15
Pathway	(KEGG) Regulation of autophagy	Positive (0.31)	P3, P7, and P15
Pathway	(GOBP) cAMP metabolic process	Positive (0.52)	P3, P7, and P15
TF ^b^	SOX9	Positive (0.35)	P3, P7, and P15
TF ^b^	SMAD3	Positive (0.20)	P3, P7, and P15
TF ^b^	RARG	Positive (0.28)	P3, P7, and P15
TME ^c^	Cancer-associated fibroblast	Positive (0.44)	P15
TME ^c^	Mast cell	Positive (0.36)	P15
TME ^c^	Regulatory T cell	Positive (0.27)	P15

* Correlation refers to the r obtained from the Pearson correlation coefficient analysis between the GRC score and each feature. ^a^ This analysis was conducted using GSVA (gene set variation analysis). ^b^ This analysis was conducted using DecoupleR. ^c^ This analysis was conducted using xCell. HM, Hallmark; KEGG, Kyoto Encyclopedia of Genes and Genomes; GOBP, Gene Ontology of biological pathway.

**Table 3:** Correlation analysis between GRC score and biological features, including pathways and transcription factors in the P3 and P7 phases.

Methods	Biological Pathways	Z-Diff (Rank) *	Active Phase
Pathway ^a^	(HM) PI3K AKT MTOR signaling	0.26 (3/50)	P3 and P7
Pathway ^a^	(KEGG) PPAR signaling	0.34 (13/186)	P3 and P7
Pathway ^a^	(GOBP) Monoacylglycerol metabolic process	0.60 (7/481)	P3 and P7
Pathway ^a^	(GOBP) Succinyl CoA	0.53 (12/481)	P3 and P7
TF ^b^	PPARG	0.51 (8/300)	P3 and P7
TF ^b^	PPARGC1A	0.31 (11/300)	P3 and P7
TF ^b^	HDAC7	0.80 (2/300)	P3 and P7
TF ^b^	MECP2	0.70 (3/300)	P3 and P7

* Z-diff refers to the difference in Z-value between the P3 and P7 and the remaining stages. * Rank was evaluated based on the Z-diff value for each relevant analysis result. ^a^ This analysis was conducted using GSVA (gene set variation analysis). ^b^ This analysis was conducted using DecoupleR. HM, Hallmark; KEGG, Kyoto Encyclopedia of Genes and Genomes; GOBP, Gene Ontology of biological pathway.

**Table 4:** Correlation analysis between GRC score and biological pathways in P15 phase.

Methods	Biological Pathways	Correlation (R) *	Active Phase
Pathway ^a^	(HM) WNT Beta-catenin signaling	Positive (0.51)	P15
Pathway ^a^	(KEGG) Focal adhesion	Positive (0.48)	P15
Pathway ^a^	(KEGG) MAPK signaling	Positive (0.43)	P15
Pathway ^a^	(HM) TGF Beta signaling	Positive (0.38)	P15
Pathway ^a^	(HM) NOTCH signaling	Positive (0.34)	P15
Pathway ^a^	(KEGG) ECM–receptor interaction	Positive (0.34)	P15
Pathway ^a^	(KEGG) VEGF signaling	Positive (0.23)	P15
Pathway ^a^	(HM) EMT	Positive (0.26)	P15

* Correlation refers to the r obtained from the Pearson correlation coefficient analysis between the GRC score and each feature. ^a^ This analysis was conducted using GSVA (gene set variation analysis). HM, Hallmark; KEGG, Kyoto Encyclopedia of Genes and Genomes.

**Table 5:** Correlation analysis between secreted factor-related biological pathways and GRC score.

Methods	Biological Pathways	Correlation (R) *	Roles in TME
Pathway ^a^	(GOBP) cAMP metabolic process	Positive (0.52)	Immune suppression
Pathway ^a^	(KEGG) Arachidonate metabolic process	Positive (0.30)	Immune suppression
Pathway ^a^	(GOBP) Adenosine metabolic process	Positive (0.30)	Immune suppression

* Correlation refers to the r obtained from the Pearson correlation coefficient analysis between the GRC score and each feature. ^a^ This analysis was conducted using GSVA (gene set variation analysis). KEGG, Kyoto Encyclopedia of Genes and Genomes; GOBP, Gene Ontology of biological pathway.

**Table 6:** Representative therapeutic strategies and clinical examples targeting resistance-associated processes.

Category	Therapeutic Strategy	Representative Agent	Clinical Trial	Phase
Non-genetic plasticity	EZH2 inhibition + ICB	Tazemetostat + pembrolizumab	NCT03854474	Phase I/II
Non-genetic plasticity	HDAC inhibition + ICB	Entinostat + pembrolizumab	NCT03765229	Phase II
Metabolic reprogramming	OXPHOS inhibition	IACS-010759	NCT03291938	Phase I
Metabolic reprogramming	Metabolic drug repurposing	Metformin + imvastatin	NCT02360618	Phase II
TME remodeling	Chemotherapy + ICB	Durvalumab + gemcitabine/cisplatin	NCT03732677	Phase III
TME remodeling	TGF-β inhibition + ICB	Linavonkibart + pembrolizumab	NCT04291079	Phase I
TME remodeling	CD73 blockade + ICB	Oleclumab + durvalumab	NCT03773666	Phase I
TME remodeling	FAP-directed therapy	[177Lu] Lu-FAP-2286	NCT04939610	Phase I/II

ICB, immune checkpoint blockade; OXPHOS, oxidative phosphorylation; TME, tumor microenvironment.

## Data Availability

No new experimental data were generated in this study. Secondary analyses were performed using previously published GRC model-derived transcriptomic resources (GSE190636 and GSE210954) and the publicly available bladder cancer cohort (GSE13507).

## Abbreviations

The following abbreviations are used in this manuscript:

GRC	Gemcitabine-Resistant Cancer
DTP	Drug-Tolerant Persister
EMT	Epithelial–Mesenchymal Transition
OXPHOS	Oxidative Phosphorylation
TCA	Tricarboxylic Acid Cycle
ROS	Reactive Oxygen Species
TME	Tumor Microenvironment
ECM	Extracellular Matrix
CAF	Cancer-Associated Fibroblast
TAM	Tumor-Associated Macrophage
MDSC	Myeloid-Derived Suppressor Cell
Treg	Regulatory T cell
GSVA	Gene Set Variation Analysis
HM	Hallmark
KEGG	Kyoto Encyclopedia of Genes and Genomics
GOBP	Gene Ontology of Biological Pathway
